# Solar Disinfection of MODS Mycobacterial Cultures in Resource-Poor Settings

**DOI:** 10.1371/journal.pone.0001100

**Published:** 2007-10-31

**Authors:** Ruvandhi Nathavitharana, Jorge Coronel, David A. J. Moore

**Affiliations:** 1 Department of Infectious Diseases and Immunity and Wellcome Centre for Clinical Tropical Medicine, Faculty of Medicine, Imperial College London, London, United Kingdom; 2 Laboratorio de Investigación de Enfermedades Infecciosas, Universidad Peruana Cayetano Heredia, San Martín de Porras, Lima, Perú; 3 Asociación Benéfica PRISMA, San Miguel, Lima, Perú; Albert Einstein College of Medicine, United States of America

## Abstract

**Introduction:**

Safe disposal of TB culture material in which the infectious burden of clinical samples has been greatly amplified is an important challenge in resource-limited settings. The bactericidal capacity of solar cookers has been demonstrated previously for conventional bacteria and contaminated clinical waste. We investigated the use of a simple solar cooker for the sterilization of mycobacterial broth cultures from the microscopic observation drug susceptibility assay (MODS).

**Methods:**

Simulated TB culture materials were prepared by inoculating 24-well MODS plates with 500 µL of a known concentration of *Mycobacterium bovis* BCG. In a series of experiments, samples were simultaneously placed inside a box-type solar cooker and control box and removed at timepoints between 15 minutes and 6 hours. Quantitative cultures were performed using retrieved samples to determine sterilization effect.

**Results:**

All cultures from the control box were positive at or within 1–4 logs of inoculation concentration. Simulated culture plates at concentrations from 10^3^colony-forming-units (CFU)/ml to 10^7^ CFU/ml were completely sterilized after only one hour of cooker exposure, at temperatures between 50–102°C. At 10^9^ CFU/ml (far in excess of diagnostic cultures), it was only possible to recover mycobacterial growth in plates removed after 15 minutes. By 30 minutes all plates were effectively sterilized.

**Discussion:**

Solar disinfection provides a very effective, safe and low-cost alternative to conventional equipment used for disposal of mycobacterial culture material. Effect of climatic conditions and optimal operating procedure remain to be defined.

## Introduction

The re-emerging global epidemic of tuberculosis (TB) is responsible for almost 2 million deaths annually, although it is a curable disease[1]. The majority of disease morbidity and mortality is felt in the developing world, where facilities for both diagnosis and treatment may be limited. In addition to the reinforcement and scale-up of existing tools, new diagnostic technologies and strategies are regarded as essential elements for accelerated global TB control.

The low-tech Microscopic Observation Direct Susceptibility assay (known as MODS) was developed specifically with resource-limited settings in mind. MODS facilitates efficient detection of *Mycobacterium tuberculosis* from sputum samples and rapid, reliable direct drug susceptibility testing (DST) through microscopic observation of liquid TB cultures in a median of 7 days[2], [3]. Evaluation to date has focused on the performance of MODS in improving diagnosis but an important issue for every TB laboratory is safe disposal of the amplified mycobacterial load generated by liquid cultures. This aspect of MODS implementation has not been previously investigated, though it is clearly of fundamental importance in the field.

The bactericidal effect of ultraviolet (UV) irradiation is well documented and solar radiation has been successfully used to treat contaminated drinking water[4]. The use of a simple box type cooker for solar disinfection has been previously reported for microbiological materials contaminated with other bacteria[5], but has not been investigated for *Mycobacterium tuberculosis* (MTB). It is known that MTB is susceptible to both UV radiation[6] and heat, probably as little as 80°C for 20 minutes (which is less than standard autoclaving temperatures)[7]. Though resources for laboratory equipment such as autoclaves and incinerators may be limited in high TB burden developing countries, many have an abundance of solar energy (sunlight). Solar cookers are in widespread use across the world and provide a less expensive and less ecologically harmful alternative for food preparation than conventionally fired ovens[8]. We investigated whether disinfection using a solar, cooker, could potentially provide a low-cost yet effective alternative for safe decontamination of diagnostic MTB cultures generated by MODS.

## Methods

### Solar cooker

**Figure 1:**
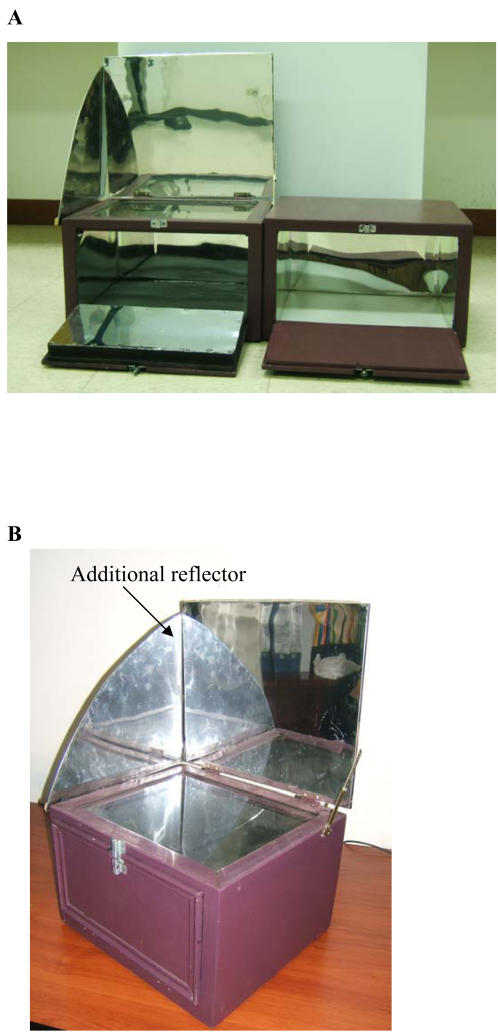
Box type solar cooker used in this study (A) original design (left) with control box (right) and (B) final design with additional reflector.

The conventional box-type solar cooker (external dimensions 25×35×40 cm) was constructed of wood with a glass lid, internal walls of aluminium, a black base and a hinged front access door; an additional external reflector was added after the first two experiments (Figure 1). The cooker cost $50.

### Preparation of samples

Simulated TB culture materials were prepared for each experiment by inoculating each well of a 24-well MODS plate with 500 µL of a known concentration of *Mycobacterium bovis bacillus Calmette-Guerin* (BCG). BCG was used for reasons of safety and because BCG has been shown to be of similar susceptibility to UV radiation as wild-type *M tuberculosis* [9]. Suspensions of BCG were prepared from log dilutions of MacFarland 1.0 turbidity standard, containing 3×10^8^ colony-forming-units (CFU)/mL, using Middlebrook 7H9+10% OADC (oleic acid-albumin-dextrose-catalase) nutrient broth. Tween 80 (1%) was used to prevent cell clumping. Three different dilutions were prepared for each experiment.

### Experiments

On each day of experimentation, 24-well plates were inoculated such that groups of 8 wells each contained one of three different concentrations of BCG (ranging from 10^3^ to 10^9^/mL). Experiments with the cooker were performed during the southern hemisphere winter month of August 2006, outdoors in the town of Chosica (20 miles east of Lima, 11°55′ south of the equator). Plates (in plastic ziplock bags within sealed autoclave bags) were placed in the cooker, which had been positioned facing the direction of the sunlight.and allowed to pre-warm to at least 80°C. One plate, containing three groups of 8 wells with differing concentrations of BCG, was removed at each of a set of designated time points over the course of a day of experimentation. In order to test the capacity of the cooker extensively, we varied the time points at which plates were removed between different experiments on different days; for example, either 2, 4, 6 hours; or 1, 2, 3 hours; or ¼, ½, 1, 2 hours. Retrieved plates were stored in a cool box with icepacks until processing later the same day in the laboratory. As intervention-free controls, identical sets of plates were placed in a control box kept in the shade which had the same dimensions as the cooker but lacked a glass lid and reflective walls, and were removed at each of the same time points. Measurements of temperature using a thermocouple and light intensity using a radiation meter were taken at the beginning and end of each experiment and at each time point.

### Cultures

The contents of the wells for each concentration were harvested from each plate using a microliter pipette and transferred to a 15 ml Falcon tube. Serial dilutions of the pooled harvested samples in Middlebrook 7H9 medium with OADC were made at 1∶1, 1∶10, 1∶100 and 1∶1000 for quantitative culture. Samples were inoculated into MODS plates as previously described [2], [3], incubated at 37°C and read under an inverted light microscope at x40 magnification on days 6, 8, 10, 14 and 21. The time to positivity and the number of CFU recovered from each sample at each time point were recorded. The numbers of mycobacteria in the samples were determined by limiting dilution in liquid media, with microscopic observation for growth of characteristic groups of bacteria. Effective sterilization was defined as plates from which no surviving mycobacteria could be recovered even after prolonged MODS culture of six weeks.

### Analysis

Data were collected in a Microsoft Excel 2003 database. The absolute and relative reductions in CFU counts achieved in the cooker were compared to the control culture plates.

## Results

**Figure 2:**
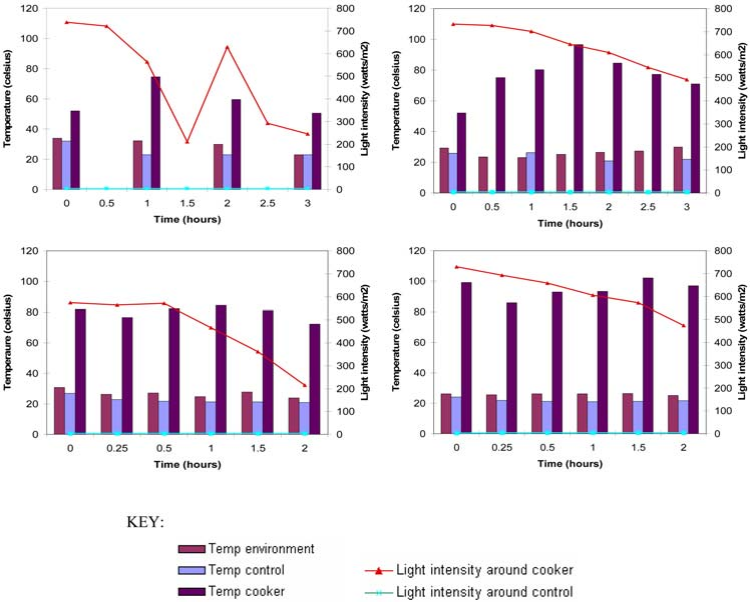
Variation in light intensity (at cooker and control sites) and temperature (environmental and inside control and solar cooker boxes) in four illustrative experiments.

Despite considerable within-day and between-day variation in light intensity environmental temperature was fairly stable (figure 2). Variability in light intensity had relatively little effect upon the existing temperature within the solar cooker. With the addition of the additional reflector, the length of pre-warming time required for the cooker to reach 80°C was between 15 and 30 minutes. The highest temperature attained inside the cooker was 102°C.

**Figure 3:**
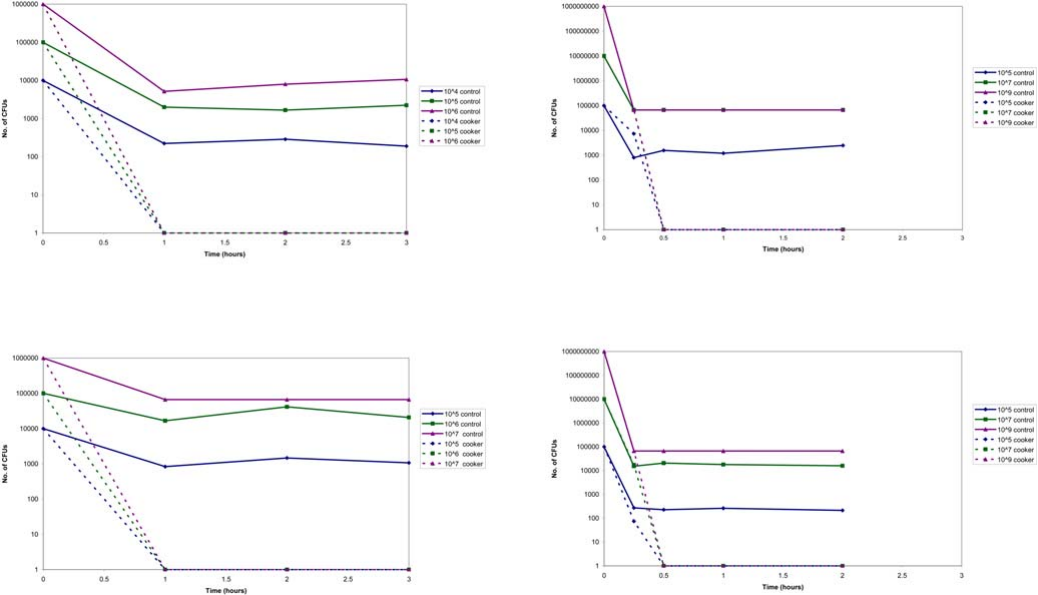
The effect of time spent in solar cooker or control box upon mycobacterial recovery (in CFU/ml) from exposed (cooker) and unexposed (control) plates. Four different data sets are demonstrated above. Note: the baseline CFU count relates to the inoculum; all other CFU counts relate to harvested samples.

In all experiments the pooled cultures recovered from plates placed in the control box yielded positive cultures at or within 1–2 logs of inoculation concentration at concentrations from 10^3^ CFU/ml to 10^6^ CFU/ml and within 2–4 logs of inoculation concentration at concentrations from 10^7^ CFU/ml to 10^9^ CFU/ml (figure 3). In initial experiments simulated culture plates at test concentrations from 10^3^/ml to 10^7^/ml were all completely sterilized by one hour of cooker exposure (figure 3a and b, data only shown for 10^5^ CFU/ml, 10^6^ CFU/ml and 10^7^ CFU/ml). In subsequent experiments utilizing higher concentrations of inoculum and shorter cooker time periods all plates were effectively sterilized by 30 minutes in the cooker (figure 3c and d). Even at 10^9^ CFU/ml (far in excess of diagnostic TB culture concentrations), mycobacterial growth was only detected in plates removed after 15 minutes; all plates removed at later timepoints were rendered culture-negative.

## Discussion

This study has demonstrated the remarkable capacity of a simple, low-tech, low-cost solar cooker for sterilizing heavily contaminated mycobacterial culture materials. At all concentrations of mycobacteria, plates left in the cooker were effectively sterilized after a maximum exposure time of 1 hour, which was a shorter time period than had been expected, increasing the practicability of this method as an efficient alternative to conventional systems such as autoclaves and incinerators. These findings are consistent with previous reports on sterilization of other infectious material using solar cookers^5^. We tested the capacity of the solar cooker by using higher concentrations of mycobacteria than would be expected to be found in MODS diagnostic culture plates and shortening exposure times. The MODS methodology is much more sensitive and rapid than other culture techniques with a sensitivity of 97.8% in comparison to 89.0% for automated mycobacterial culture and 84.0% for culture on Löwenstein Jensen medium and a median time to culture positivity of 7 days, in comparison to 13 days and 26 days respectively^3^. Samples are never positive after 30 days even for patients receiving TB therapy. Nonetheless, to ensure that no late positive cultures were missed, we retained the MODS cultures of retrieved materials for six weeks (normal maximum culture time is 21 days) to cover the unlikely possibility of extremely late recovery, so we are confident that ascertainment was complete.

Despite large fluctuations in light intensity, both environmental temperatures and those within the cooker remained fairly consistent. However these experiments were deliberately performed in a sunny environment and the effect of cloud cover and seasonal variation in ultra violet light intensity upon sterilization capacity should be examined. Since we placed plates within opaque autoclave bags for biosafety, it must be assumed that the sterilization effect in these experiments was heat-mediated and not related to ultraviolet light induced killing. Whether sterilization would be further enhanced if transparent bags were used warrants further study.

We observed a significant reduction in recoverable mycobacteria at the first time point in all control experiments though at subsequent sampling points no further reductions in CFU over time were noted. These findings highlight the crucial importance of inclusion of a non-exposed control arm in this study design and we believe are explained by the consistently challenging technical difficulty in harvesting and pooling culture material samples.

The cost of this simple box type solar cooker was approximately US$50, in comparison with laboratory autoclaves, which require a reliable electric supply and may cost upward of US$2000 with significant running and maintenance costs. Larger cookers are available and further modifications such as the use of multiple reflectors, parabolic mirrors or additional electrical heating connections can increase heat production.

We have demonstrated that solar disinfection provides a very effective and low-cost alternative to conventional equipment used for sterilization of mycobacterial culture material. Further study with *M. tuberculosis* should determine the effect of differing climatic conditions such as environmental temperature, seasonal variation in ultraviolet light intensity and humidity. We believe that this method is particularly applicable to resource poor settings where the disease burden of TB is highest.
